# Driving CARs to BARs: The Winding Road to Specific Regulatory T Cells for Tolerance

**DOI:** 10.3389/fimmu.2021.742719

**Published:** 2021-09-06

**Authors:** David W. Scott

**Affiliations:** Department of Medicine (MED), Uniformed Services University of the Health Sciences, Bethesda, MD, United States

**Keywords:** chimeric antigen receptor, tolerance, autoimmunity, hemophilia, allergy, EAE (experimental autoimmune encephalitis), regulatory T cell (Treg)

## Abstract

Chimeric antigen receptor (CAR) transduced T cells have significantly improved cancer immunotherapy. Similarly, engineering regulatory T cells (Treg) with specific receptors to endow specificity and increase efficacy of Tregs holds great promise for therapy of a variety of adverse immune responses. In this review, we focus on our approaches using retroviral transduction of specific T-cell receptors, single chain variable fragments (scFv) or antigen in models of monogenic diseases, autoimmunity and allergy. The advantages of each of these for different targets diseases are discussed as well as their potential for clinical translation.

## Introduction

Nearly five decades ago, Gershon and colleagues at Yale proposed that immune responses can be controlled by a subset of T cells called “suppressor cells” that downregulate other lymphoid cells (1). At that time, immunologists lacked the reagents to fully characterize these suppressors and their mode of action other than cell mixing experiments. Ironically, the latter approach still remains a *sine qua non* to demonstrate their efficacy. Armed with flow cytometry and molecular biology approaches, including the discovery of FOXP3 and its association with human immunodeficiency and autoimmune diseases (2–5), suppressor cells were replaced by a well-defined entity, “regulatory T cells” (Tregs) (2, 6). Similarly, these cells have the ability to suppress a variety of immune responses *in vitro* and *in vivo*.

Clinical trials with expanded Tregs were initiated over a decade ago in transplantation and autoimmunity, as summarized in Romano et al. (7). While these cells have been used safely in multiple clinical trials, they are polyclonal and the frequency of specific Tregs is very low. Expanded Tregs express a broad repertoire of specificities, and have the potential to be non-specifically immunosuppressive (8). To overcome the latter issue and the rare frequency of specific T cells, our lab has focused the use of *specifically engineered* Tregs to suppress adverse immune responses in monogenic diseases, autoimmunity and allergy. Our studies are based on the seminal studies of Eshhar and colleagues, who first demonstrated expression of specific receptors in T cells (9). These pioneering studies have been successfully applied using single chain antibody fragments (scFv) in cancer immunotherapy worldwide (10, 11). A major example is the use of anti-CD20 engineered human T cells in the successful treatment of leukemia (10). The first successfully use of specific Tregs used expanded FoxP3-expressing transgenic T cells in an autoimmune model of multiple sclerosis (12). Since that time, multiple laboratories have made significant contributions by engineering specificity into murine and/or human Tregs (13–25). The purpose of this manuscript is to highlight our approaches in the context of this rapidly developing field focusing on targeting specific adverse responses.

**Figure 1:**
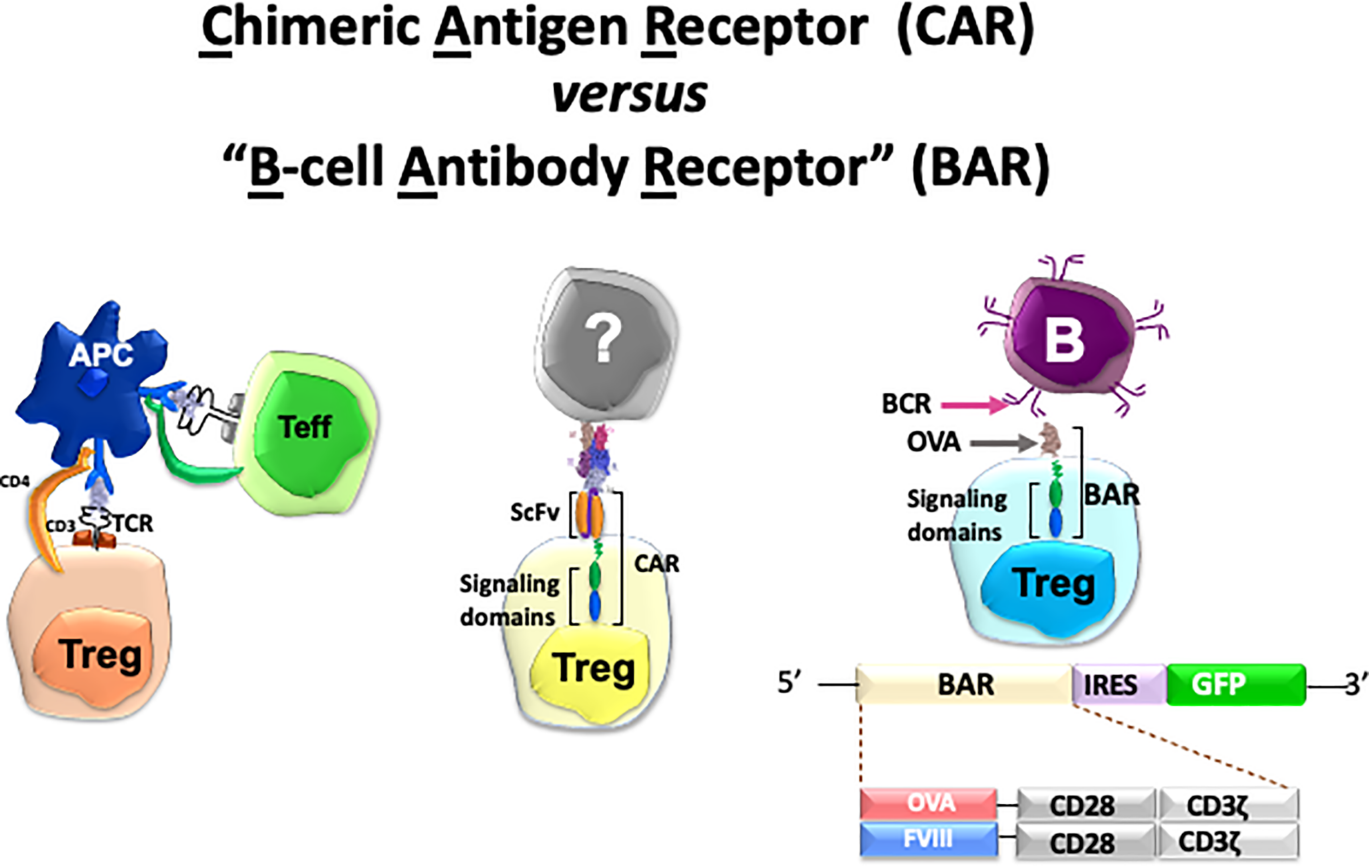
Cartoon of three types of specific Tregs and potential targets used in our lab. See **Table 1** for details.

Specificity in our lab has been achieved by engineering Tregs to express receptors that can recognize the targets of adverse immune responses. Thus, we have applied this protocol in autoimmunity, hemophilia A and allergy. To achieve this goal, we have used retroviral transduction of cloned T-cell receptors (TCR’s), scFv’s or antigen domains in thymic-derived human natural regulatory T cells (see **Figure 1**). In this review, we describe the basic principles and progress in each of these efforts by ourselves in three disease models to achieve the ultimate goal of modulating adverse human diseases (26–31). We also summarize the advantages and disadvantages of these approaches below and in **Table 1**. We have used retroviral transduction of Tregs with CD3 and CD28 signaling domains as the basic version; efforts to modify the signaling process has been reviewed by others (16, 32).

**Table 1:** Advantages and disadvantages of engineered Treg approaches.

Gene modified Treg	Specificity (Target antigens)	Disease model	Cellular targets	Advantages	Disadvantages
T-cell receptor (TCR)	MHC-restricted epitopes (Factor VIII; Myelin basic protein)	Hemophilia (FVIII knockout) mice (26) Multiple sclerosis (EAE) (27)	Antigen-presenting cells	Suppression of CD4 effector proliferation and cytokine production; Suppression of antibody formation; Bystander effect for suppression; Not affected by circulating antibody	HLA-restricted; Need to clone different TCRs; may be affinity dependent
Single change Fv chimeric receptor (CAR)	Conformational epitopes (FVIII; MOG/MBP)	Hemophilia (FVIII knockout) mice (28) Multiple sclerosis (EAE) (18)	Cell surface membrane antigens	Suppression of CD4 effector proliferation and cytokine production; Suppression of antibody formation; Bystander effect for suppression; Not affected by circulating antibody	Need to recognize intact conformational epitope/domain
B-cell antigen receptor (BAR)	Antigen-specific B cell Fcε receptor on Mast cells	Hemophilia (FVIII knockout) mice (29, 30) Allergy (OVA; Peanut) (31)	B-cell receptor; IgE in Fcε receptor	Suppression of antibody formation; Bystander effect for suppression of antibody formation;?Not affected by circulating antibody	Unknown bystander effect for suppression of allergy

## Choosing Targets, Choosing Receptors, Establishing Collaboration

Typical pharmacologic therapies for adverse immune responses are broadly immunosuppressive. An issue to achieve any specific tolerogenic therapy is the choice of targets. Indeed, many adverse responses have multiple targets, with no unique or specific antigen being attacked. Moreover, any targeted antigen may have a variety of T-cell and B-cell epitopes. Thus, the challenge in rendering Treg cell-based therapy specific depends on multiple factors: knowing the target antigen and having the appropriate receptors. This is easier in monogenic diseases wherein the target antigen is known as patients lacking this protein often produce antibodies to the missing antigen when treated therapeutically. Patients are not tolerant to the missing protein and treat the therapeutic protein as a foreign antigen. Such is the case in hemophilia A (HA), an X-linked disease with a frequency of 1 in 5000 males in which patients have mutations in the *F8* gene encoding pro-coagulant Factor VIII (FVIII). Approximately 30% of HA patients develop high-titer neutralizing antibodies against therapeutic FVIII following repeated infusions of this needed protein that inhibit the function of this life saving therapy (33, 34). Most of these inhibitors block FVIII activity by binding to two immunodominant domains, called C2 and A2, which are important for FVIII’s pro-coagulant activity.

In 2012, Yongchan Kim joined my lab after a successful post-doctoral fellowship with Ethan Shevach, a Treg expert at NIH. At the same time, Kathleen Pratt joined the faculty in our department. Dr. Pratt had cloned several T-cell lines from HA patients (35, 36), and one of these T cell clones, called 17195, recognized an HLA-restricted peptide in the C2 domain of FVIII, residues 2194-2210 (35, 37). With a determination of the TCR V regions, Yongchan then inserted them into a retroviral vector and used the vector to transduce FACS-purified human Tregs (see ref. (27) for detailed methods). Purified Tregs were CD25^high^ and CD127^low^ and typically expressed FOXP3 and Helios transcription factors. Although less than 20% of the initially transduced Tregs expressed the 17195 T-cell receptor (TCR), this population expanded upon stimulation with the FVIII 2194-2210 peptide on HLA DR1 antigen-presenting cells (26). This stimulation also led to an increased expression in FOXP3 and Helios transcription factor, markers that confirmed the expansion of Tregs in culture. Importantly, these expanded 17195-expressing Tregs suppressed the proliferation and cytokine production by FVIII-specific T effector cells even more effectively than polyclonal Tregs *in vitro*. Moreover, these expanded human 17195-expressing Tregs also suppressed the secondary antibody response of *murine* HLA-DR1 transgenic spleen cells stimulated with FVIII *in vitro*. Overall, human 17195-expressing Tregs were able to effectively suppress the antibody response to FVIII *in vivo*, even across a xenogeneic barrier, although they were rejected within two to three weeks (26). Notably, while human 17195-expressing Tregs were specific for a single peptide in the C2 domain of FVIII, they suppressed antibody formation against the multiple epitopes in the entire FVIII protein. This suggested that suppression, while “specific” for a single FVIII peptide, may have bystander effects on the response to other epitopes in FVIII locally, presumably at the level of the antigen-presenting cells.

TCR engineered Tregs are MHC-restricted, which limits their utility to HLA-matched donor recipient combinations and limited repertoires. To generate antigen-specific Tregs that were not MHC-restricted, Jeong-Heon Yoon collaborated with Christoph Königs and Anja (ne’ Naumann) Schmidt in Frankfurt. Anja had isolated several single chain (sc)Fv’s that recognized FVIII domains (38) and cloned them into our standard retroviral vector for expression in Tregs. The human Tregs transduced with retroviral vector encoding scFv recognized a conformational epitope in the A2 region of FVIII (38). When these scFv-expressing human Tregs were mixed with spleen cells from mice immunized with FVIII, they suppressed the secondary response *in vitro*, and blocked antibody formation *in vivo* in hemophilic mice. Compared to TCR-engineered Tregs, the scFv-transduced Tregs suppressed the anti-FVIII immune response to the same degree at certain ratios (28). It is worth noting, then, that engineered Tregs specific for the MHC-restricted peptide in the C2 domain of FVIII and Tregs engineered to express the scFv directed at the A2 domain of FVIII both suppressed the immune response to other domains in FVIII. Thus suggesting that both types of antigen-specific Tregs exerted bystander suppression to other epitopes in the same target protein locally, but they did not non-specifically suppress the response to an unrelated antigen, *e.g.*, immunization with TNP hapten-conjugated red blood cells (28). Application of scFv-transduced Tregs in a transplant model was elegantly demonstrated in the Levings’ lab (15), thus demonstrating further application of engineered Tregs for tolerance.

## Treg Effects in Autoimmunity

T-cell responses against self-proteins can result in a spectrum of autoimmune diseases. Unlike monogenic diseases in which the target antigens are known, multiple potential antigens and epitopes may be recognized in autoimmunity. For instance, several antigens have been identified in Type 1 diabetes including islet antigens such as glutamic acid decarboxylase or insulin. In the central nervous system (CNS), myelin basic protein (MBP) or myelin oligodendrocyte glycoprotein (MOG) can be targeted in multiple sclerosis (MS). Based on our success and the bystander effect in hemophilia with an engineered TCR recognizing FVIII, we hypothesized that transduction of an MBP-specific TCR into expanded human Tregs might be an effective therapy in MS. In collaboration with Kai Wucherpfennig at Harvard, we engineered human Tregs to express the Ob2F3 TCR V regions that targeted an immunodominant MBP peptide, p85-99 (39). Ob2F3-expressing Tregs from either healthy adults or MS patients could suppress MBP-specific T effector cells, but were also able to suppress T cells with other specificities after Tregs had been activated through the TCR (27). Surprisingly, these Ob2F3-expressing Tregs which were MBP-specific ameliorated EAE in MOG-immunized DR15 transgenic mice (22). This suggested that bystander suppression *in vivo* might be associated with soluble factors, enhanced by cell contact between Tregs and effectors (27). These results indicated that engineered MBP-specific Tregs were able to suppress autoimmune pathology in EAE.

An important question was whether Tregs engineered with scFv’s will work in autoimmunity. To answer this question, Anja Schmidt provided two single chain Fv’s reactive with MBP and MOG, and we showed that these cells recognized murine CNS tissue. Alessandra Pohl in my lab then cloned these into constructs in our retroviral vector for Treg transduction. Results showed that a mixture of human Tregs expressing these CNS “specific” scFv’s were able to suppress EAE *in vivo* similar to the TCR-expressing Tregs (18); however, direct comparison of the TCR- *versus* scFv-transduced Tregs has not yet been performed. Nevertheless, these important results indicate that engineered CNS targeting CAR-Tregs have the potential to be used as a cellular therapy for MS patients.

## Possible Mechanisms of Suppression by Engineered Tregs

**Figure 2:**
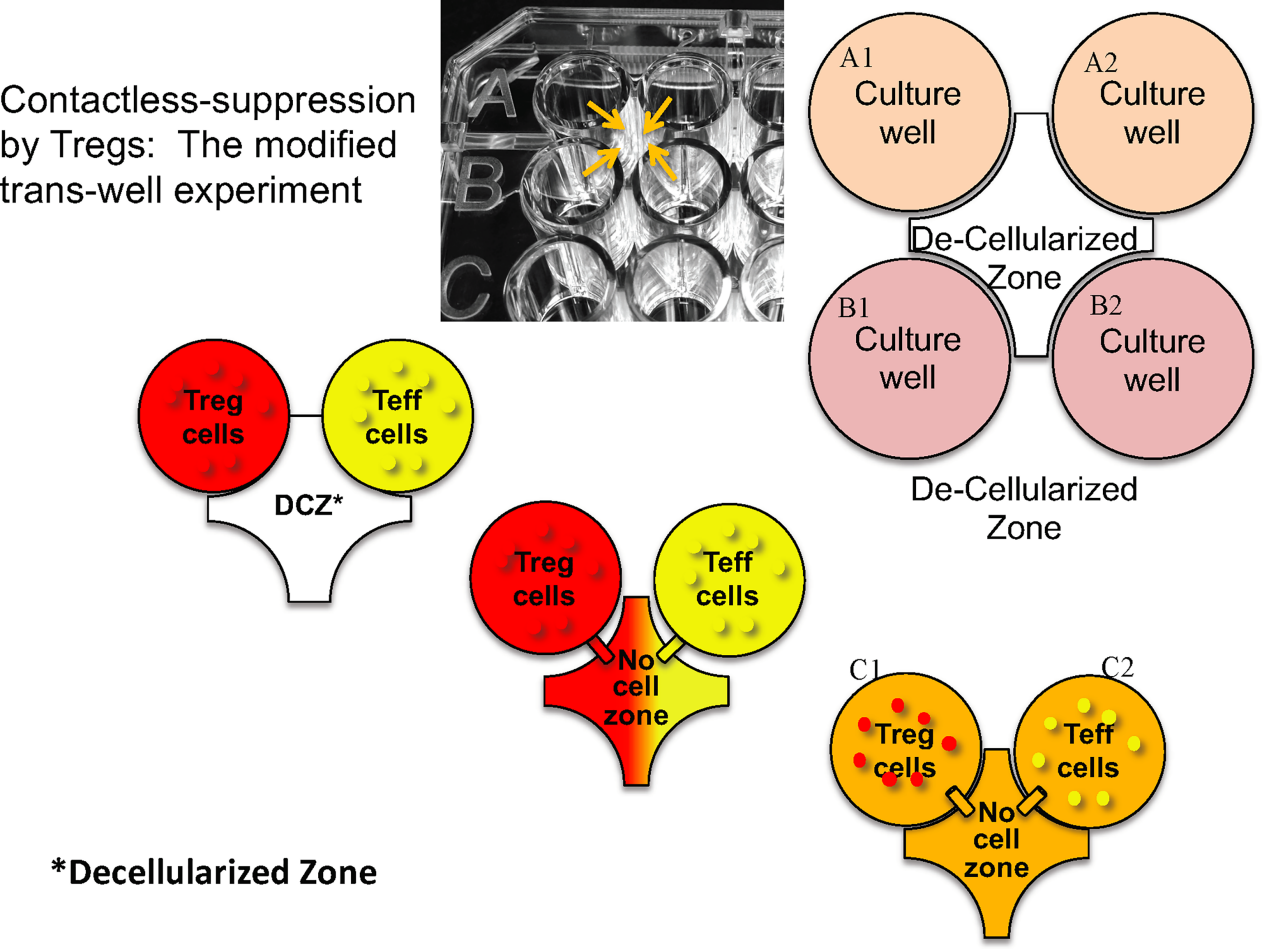
Design of microtiter plate to test whether direct cell contact is necessary for the bystander effect. Tregs and T effectors (Teff) and placed in the same or contiguous wells with a hole created above the bottom of the wells (arrows) so that fluid but no cells can migrate. “Decellularized” zone refers to the space between the four wells into which soluble products can diffuse to neighboring wells.

Using an ingenious version of a transwell system in which neighboring microtiter well liquid contents were connected *via* an opening above the cell layer (**Figure 2**), Kim showed that suppression of effector T cells in one well only occurred when the neighboring well contained *both* Tregs and effector T cells. Thus, contact between Tregs and effector cells in one well led to suppression of effector T cells not in direct contact in a neighboring microtiter well. Based on the observation that IL-2 signaling *via* interaction with CD25 can lead to STAT5 phosphorylation, we were able to show in kinetic studies that phospho-STAT5 decreased in effector CD4 T cells at the same time it was increasing in engineered Tregs (27). These data led us to propose that, similar to classical Tregs, engineered Tregs were able to capture IL-2 produced by effector T cells upon antigen presentation in the local milieu. Overall, these results indicate that the induction of engineered Treg growth led to the activation of Tregs and release of inhibitory cytokines which mediated the bystander suppressive effect observed *in vivo*.

## BAR Tregs to Target B Cells in Adverse Immune Responses

Our results suggested that specific Tregs may block adverse immune responses at the level of the antigen-presenting cells. While we knew that adverse inhibitory antibody responses that can occur in monogenic diseases were highly T-helper cell dependent, the culprits were alloreactive B cells. We reasoned that generating specific T cells expressing antigen should be recognized by (or recognize) B cells *via* their immunoglobulin receptors. Moreover, *cytotoxic* CAR CD8 T cells could kill specific B cells recognizing the expressed antigen as shown in pemphigus by the Payne group at Penn (20). Kalpana Parvathaneni in my lab, engineered human and murine cytotoxic T cells with FVIII antigen to target the B-cell antibody receptor (BAR) and showed that these BAR-engineered T cells were capable of killing FVIII-reactive B-cell hybridomas *in vitro* and *in vivo* (40). In addition, adoptive transfer of FVIII A2- and C2-BAR CD8 T cells significantly reduced the anti-FVIII antibody formation in hemophilic mice. These data suggest that BAR-engineered T cells are a promising approach for future prophylactic treatment for patients with severe hemophilia A who are at high risk of developing inhibitors.

Aihong (Allan) Zhang in the lab also demonstrated human BAR Tregs, which express only the C2 domain of FVIII, suppressed the secondary response of spleen cells from FVIII-immunized mice *in vitro*, thus confirming the bystander effect mediated by engineered Tregs (29). Moreover, FVIII-BAR Tregs also suppressed the primary response *in vivo*, and reduced priming to FVIII as evidenced by lower secondary antibody titers upon boosting. It is important to note that FVIII antigen domains on the surface of Tregs (or CD8 T cells) might be blocked by circulating antibody to immunodominant domains in immunized mice, thus competing with B cell targeting. However, this did not appear to be a major problem at least with low titered antibodies, as was shown in collaboration with Shiva Venkatesha (19), as well as by the Payne group in their model (20). Moreover, crosslinking the expressed domains on human T cells may even lead to expansion of these T cells.

## Can BAR Tregs Work in Allergy Models?

Based on the success of BAR Tregs in hemophilia, we sought to test them in an allergy model, the IgE response to ovalbumin (OVA). We hypothesized that expression of OVA in Tregs would target the OVA-specific B cells as was done for FVIII-specific B cells in hemophilia. Maha Abdeladhim immunized mice with OVA in alum, eliciting both IgE and IgG responses, and treated OVA-primed mice with OVA-BAR Tregs. Forty-eight hours later, mice were challenged with a high dose of OVA intravenously to induce anaphylaxis as measured by an acute drop in body temperature of 2-6°C. Results showed that OVA-BAR Tregs, human or mouse, blunted this temperature drop, an effect that persisted for at least one month (31). Surprisingly, no significant drop in IgE titer in primed and Treg-treated mice was observed within two weeks. However, we predict that pretreatment with BAR Tregs would prevent sensitization as it does in the hemophilia model.

**Figure 3:**
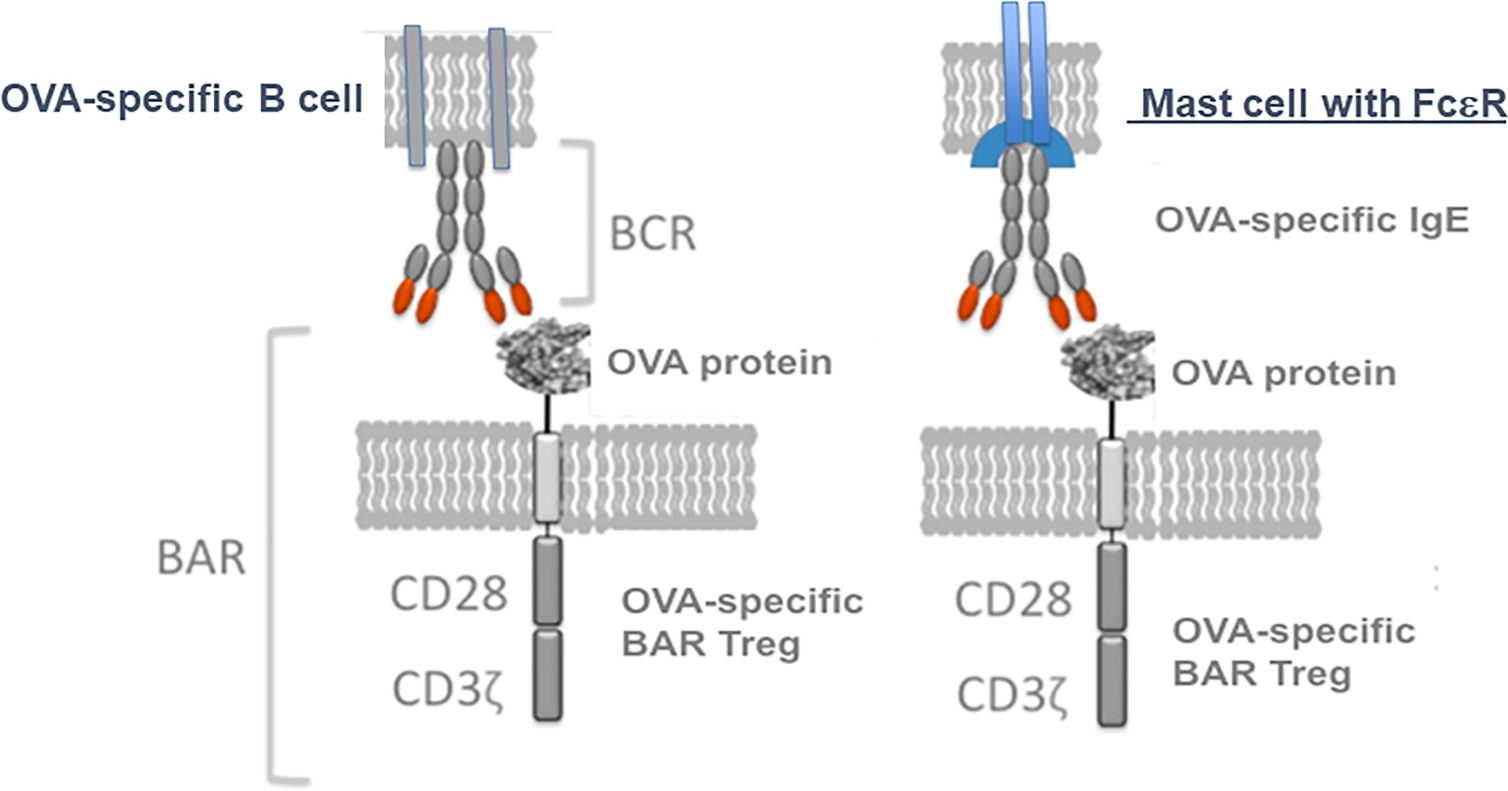
Model for BAR Tregs interaction not only with the immunoglobulin receptor on B cells, but also Ig(E) bound *via* FcεR on mast cells. Tregs expressing an antigen like ovalbumin (OVA) can interact with either specific B cell IgM (left, the BCR) or IgE bound to mast cells (right, in sensitized individuals).

We then investigated whether OVA-BAR Treg treatment targeted mast cells loaded with IgE (see **Figure 3**, right side). To this end, Maha passively sensitized *naive* Balb/c mice with anti-OVA IgE and then injected mice with OVA-BAR Tregs. To our surprise and pleasure, these OVA-BAR Tregs were very effective at blocking passive anaphylaxis, thus implicating mast cells as an important target in this system (31). Notably, BAR Tregs alone did not cause detectable release of allergic mediators despite expressing the target antigen on their surface. While the mechanism for BAR Tregs blocking anaphylaxis but not eliciting a reaction *per se* is unknown, we currently attribute this to stoichiometric differences in antigen doses. Because mast cells in patients are loaded with IgE of diverse specificities, we are currently investigating whether specific BAR Treg activity can “desensitize” to other antigens for which the mast cells are sensitized, using a peanut allergy model.

## Summary and Conclusions

**Figure 4:**
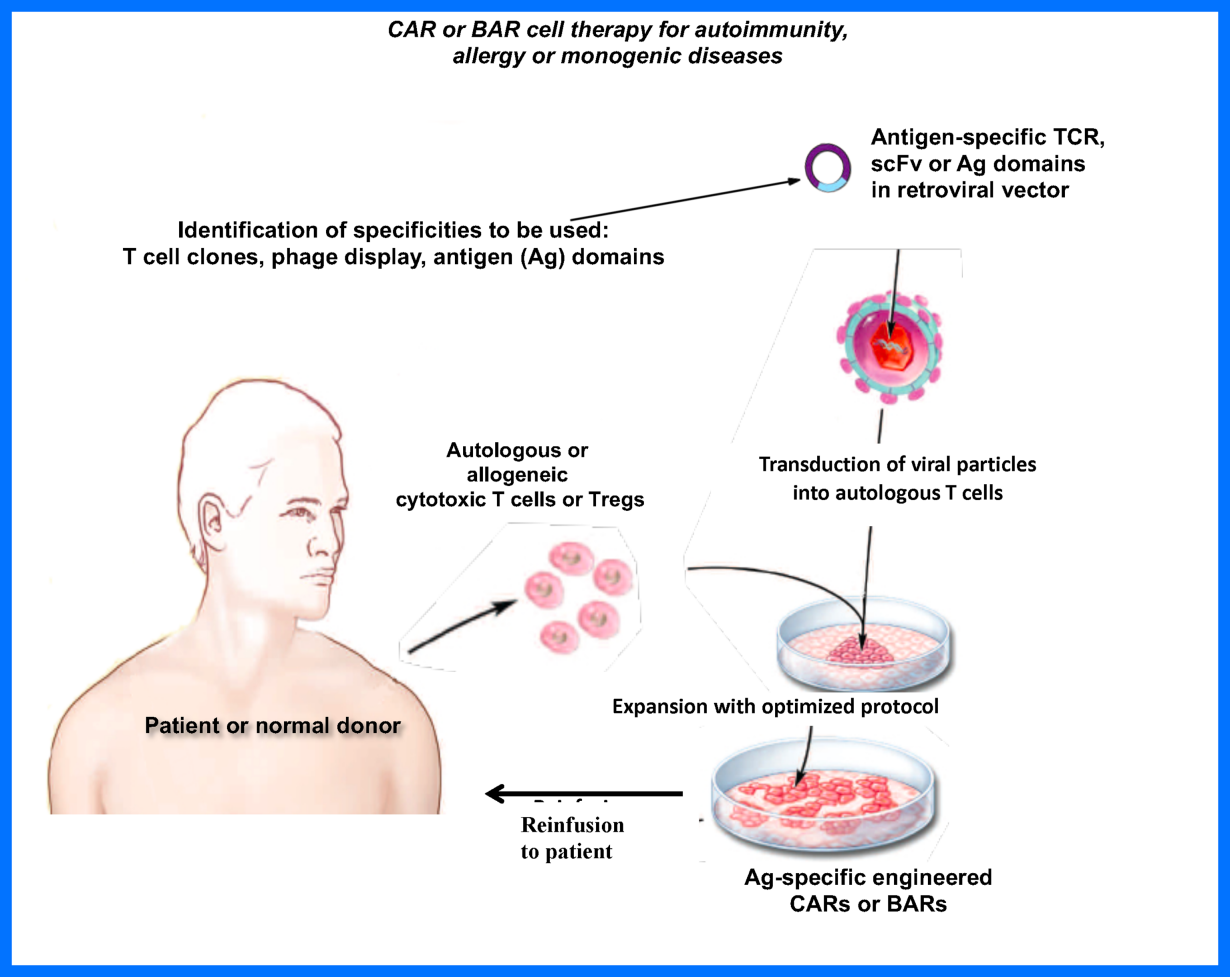
Proposed engineered Treg therapy in patients.

The studies in my lab and others (13, 15, 16) have demonstrated that Tregs can be made specific to treat a variety of adverse immune responses. The choice of which specific receptor to employ will depend on the targeted disease. For example, TCR-engineered Tregs, while highly specific, are limited by MHC diversity and by the knowledge of the variable regions of the receptor. On the other hand, scFv (CAR)-Tregs require identification of a conformational epitope in a disease. The BAR Tregs are a choice in antibody-mediated diseases as they provide a clear target, the Ig receptors recognizing antigen. Our approaches have focused on the extracellular receptor, whereas other laboratories have attempted to increase Treg signaling, with variable success (16, 41). We hope our data will be followed up by multiple efforts to increase efficacy, as well as with clinical trials to prove their efficacy and safety in the future as depicted in **Figure 4**. Efforts to create “off the shelf” generic Tregs using CRISPR engineering should also prove fruitful. The future is bright and we hope that our approaches provide the framework for Treg treatments for adverse immune responses.

## Author Contributions

The author confirms being the sole contributor of this work and has approved it for publication.

## Author Disclaimer

The opinions expressed within are those solely of the author and do not necessarily reflect the official policy or position of the Uniformed Services University or the Department of Defense of the US government.

## Conflict of Interest

The author declares that the research was conducted in the absence of any commercial or financial relationships that could be construed as a potential conflict of interest.

## Publisher’s Note

All claims expressed in this article are solely those of the authors and do not necessarily represent those of their affiliated organizations, or those of the publisher, the editors and the reviewers. Any product that may be evaluated in this article, or claim that may be made by its manufacturer, is not guaranteed or endorsed by the publisher.
